# Peroxisome Proliferator-Activated Receptor-γ Promotes Adipogenic Changes in Growth Plate Chondrocytes In Vitro

**DOI:** 10.1155/PPAR/2006/67297

**Published:** 2006-09-20

**Authors:** Lai Wang, Yvonne Y. Shao, R. Tracy Ballock

**Affiliations:** Orthopaedic Research Center, Department of Orthopaedic Surgery and Biomedical Engineering, The Lerner Research Institute, The Cleveland Clinic Foundation, Cleveland, OH 44195, USA

## Abstract

Chondrocytes and adipocytes are two differentiated cell types which are both derived from mesenchymal cells. The purpose of this study was to investigate whether peroxisome proliferator-activated receptor-γ (PPARγ), a transcription factor involved in lineage determination during adipogenesis, is able to induce adipogenic differentiation in growth plate chondrocytes. Isolated epiphyseal chondrocytes were infected with a PPARγ adenovirus or treated with the PPARγ agonist ciglitazone. Both of these treatments resulted in lipid droplet accumulation and expression of the adipogenic markers aP2, lipoprotein lipase, and adipsin in chondrocytes. Proteoglycan matrix synthesis was decreased in the PPARγ-infected cells, as was the expression of the chondrogenic genes Col2a1 and aggrecan. Growth plate cells transfected with a PPARγ expression plasmid under the control of the collagen α1(II) promoter also demonstrated a similar adipogenic changes. Terminal differentiation of growth plate chondrocytes induced by thyroid hormone was also inhibited by overexpression of PPARγ and ciglitazone treatment, with decreased expression of alkaline phosphatase and Runx2/Cbfa1 genes. These in vitro data suggest that PPARγ is able to promote adipogenic differentiation in growth plate chondrocytes, while negatively regulating chondrogenic differentiation and terminal differentiation.

## INTRODUCTION

Longitudinal growth of the skeleton is a result of endochondral ossification that occurs at the growth plate [1]. Endochondral ossification is a multistep process that includes differentiation of mesenchymal cells into chondrocytes, cell proliferation, hypertrophic differentiation, matrix mineralization, apoptosis, vascular invasion, and eventually the replacement of the cartilage by bone.

The first step of growth plate development is the commitment of mesenchymal stem cells to the chondrogenic lineage. Mesenchymal stem cells exhibit a high differentiation plasticity. They are capable of differentiating into chondrocytes, osteoblasts, adipocytes, and other tissues of mesenchymal origin [2]. Interconversion between mesenchymal phenotypes is thought to be under control of specific transcription factors, including the Sox family in chondrogenesis [3], Runx2/Cbfa1 in osteogenesis [4], and PPAR*γ* (peroxisome proliferator-activated receptor-*γ*), and C/EBP (CCAAT/enhancer-binding protein) in adipogenesis [5].

PPAR*γ* is a key transcriptional regulator of adipogenesis [5]. PPAR*γ* is also expressed in preosteoblastic cells and is thought to play a role in regulation of bone metabolism. PPAR*γ* and PPAR*γ* activators inhibit the maturation of preosteoblastic cells to osteoblasts [6–8]. Free fatty acids activate PPARs and induce adipocyte-like differentiation of osteosarcoma cell lines [6]. Lecka-Czernik et al observed that PPAR*γ*2 negatively regulates stromal cell plasticity by suppressing expression Osf2/Cbfa1 and osteoblast-like biosynthetic activity, while promoting differentiation to adipocytes [7]. Conversely, PPAR*γ* insufficiency enhances osteogenesis through increased osteoblast formation from bone marrow progenitors. Homozygous PPAR*γ*-deficient ES cells fail to differentiate into adipocytes, but increase bone mass by stimulating osteoblastogenesis from bone marrow progenitors [8].

Transdifferentiation of chondrocytes to adipocytes has been previously reported by Heermeier et al, who observed that chondrocytes of the mouse xiphoid process undergo transdifferentiation into adipocytes in the presence of 10% fetal calf serum [9].

Based on the finding that PPAR*γ* is expressed in growth plate chondrocytes [10], as well as the evidence that PPAR*γ* is able to compete with the thyroid hormone receptor (TR) for binding to retinoic acid receptor X to inhibit growth plate cell hypertrophy [11], the purpose of this study was to investigate whether PPAR*γ* and its ligands are able to promote adipogenic differentiation and suppress chondrogenic differentiation in growth plate chondrocytes.

## MATERIALS AND METHODS

### Cell culture

Chondrocytes were isolated from the resting zone of the distal femoral growth plate of 2-day old neonatal Sprague-Dawley rats by sequential digestion in trypsin/EDTA (Invitrogen, Carlsbad, Calif) for 1 hour at 37°C, followed by 0.3% collagenase type I (Worthington, Lakewood, NJ) for 4 hours at 37°C [12]. Cells were resuspended in DMEM/F12 medium (Invitrogen) supplemented with a defined media supplement (ITS+1, Sigma, St Louis, Mo) and plated in monolayer at a density of 5×10^5^ cells/cm^2^, or in a pellet culture of 1×10^5^ cells/mL as indicated [12]. Tri-iodothyronine (T3, Sigma) at a concentration of 100 ng/mL and ciglitazone (BioMol, Plymouth Meeting, Pa) at a concentration of 5 *μ*M were added to the medium as indicated.

### Immunoblotting

Total cellular protein was extracted from chondrocytes treated with 5 *μ*M of ciglitazone using RIPA buffer [9]. An equal amount of protein was subjected to SDS-PAGE, and transferred onto nitrocellulose membranes. The blots were incubated with anti-PPAR*γ* and anti-actin (Santa Cruz Biotechnology, Santa Cruz, Calif) followed by a HRP-conjugated secondary antibody. Immunoreactive proteins were visualized by Western Blotting Chemiluminescence Luminol Reagent (Santa Cruz Biotechnology). Immunoblot bands were quantitated with Kodak 1D Image Analysis Software (Eastman Kodak Company, Rochester, NY).

### Adenovirus infection

Recombinant adenovirus carrying PPAR*γ*1 (Ad-PPAR*γ*) was kindly provided by Dr J. L. Jameson (Northwestern University Medical School, Chicago, Ill). Ad-PPAR*γ* contains mouse PPAR*γ*1 cDNA driven by the CMV promoter/enhancer with an SV40 polyadenylation sequence [13]. Ad-Gal, which contains *β*-galactosidase driven by CMV promoter, was used to evaluate the efficiency of gene transduction. Eighteen hours after plating in monolayer, growth plate chondrocytes were infected with adenoviral vectors at a multiplicity of infection (MOI) of 100. Fresh media were added 24 hours after infection and incubated for 72 hours to collect the cell protein extracts. *β*-galactosidase expression was detected in 80% of cells after 24 hours of infection with Ad-Gal. Expression of introduced PPAR*γ* genes was confirmed by immunoblot.

### Plasmid construction and transient transfection

The full-length cDNA of mouse PPAR*γ* was excised by *Asp718/NheI* digestion from pCMX-PPAR*γ* (kindly provided by Dr R. Evans, Salk Institute, La Jolla, Calif). The ends of this fragment were blunted with Klenow polymerase and ligated to a blunt-ended BamHI site in the p1757 plasmid containing the rat *α*1(II) collagen promoter (kindly provided by Dr Y. Yamada, NIDR, Bethesda, Md) [14]. The cDNA encoding the mouse PPAR*γ* was thus located downstream of the rat *α*1 (II) collagen promoter element (−977 to +110). Nucleotide sequence analysis confirmed the correct orientation of the PPAR*γ* cDNA.

Growth plate cells were transfected with 10 *μ*g of p1757-PPAR*γ* or p1757 as a negative control by lipofection (Fugene 6, Roche, Indianapolis, Ind) in the presence of 4 units/mL of hyaluronidase. Sixteen hours later, the cells were trypsinized and centrifuged to pellets cultured in DMEM/F12 plus ITS+ supplements [11].

### Histochemical staining

For the analysis of adipogenic differentiation, adipogenesis and lipid accumulation in the growth plate cells were examined by staining with Oil Red-O. After 10 days of culture, cells were washed gently with PBS followed by staining with a filtered solution of 0.5% Oil Red-O (Sigma) in 60% isopropanol for 20 minutes. After washing cells with PBS three times, cells were kept in 75% glycerol solution and observed under a phase-contrast microscope.

Alcian blue staining was used to detect chondrocyte-specific proteoglycans at 10 days of culture. Cells were stained with a 4 : 1 ratio of 0.1 M HCl/0.5% Alcian blue stock (0.5% Alcian blue in 95% ethanol) overnight at 37°C in a humidified atmosphere. Cells were then washed twice with PBS to stop reaction and once with 70% ethanol to reduce background.

For alkaline phosphatase (ALP) staining, cultured plates were rinsed with PBS at 10 days of culture, fixed in 3.7% formaldehyde at room temperature for 10 minutes, and stained in the dark for 30 minutes in a 0.1 M Tris-HCl solution (pH 8.5) containing 0.2 mg/mL of Napthol AS-MX phosphate and 0.6 mg/mL of Fast Blue BB salt (Sigma).

### Quantitative real-time RT-PCR

The expression of chondrocyte or adipocyte-specific RNA markers was analyzed using quantitative real-time RT-PCR. Total RNA was isolated from cultured growth plate chondrocytes using the RNeasy Kit (Qiagen, Valencia, Calif) 4 days after adenovirus infection or plasmid transfection. Reverse transcription was performed using random primers and Superscript III (Invitrogen). Real-time PCR reactions were conducted in an ABI Prism 7700 Sequence Detection System using SYBR Green PCR core reagents (Applied Biosystems, Foster City, Calif). The comparative C_T_ method (ΔΔC_T_ method) was utilized for relative quantitation of gene levels of expression. 18S rRNA was used as an internal control for normalization of target gene expression. The forward and reverse primers for the amplifications are listed in Table 1.

### Statistical analysis

The data for real-time PCR are represented as mean ± standard deviation. Values are assessed by one-way ANOVA with the Bonferroni post-hoc test and Student *t* test at a significance level of *P* < .05.

## RESULTS

### Ciglitazone upregulates PPAR*γ* expression in growth plate chondrocytes

**Figure 1:**
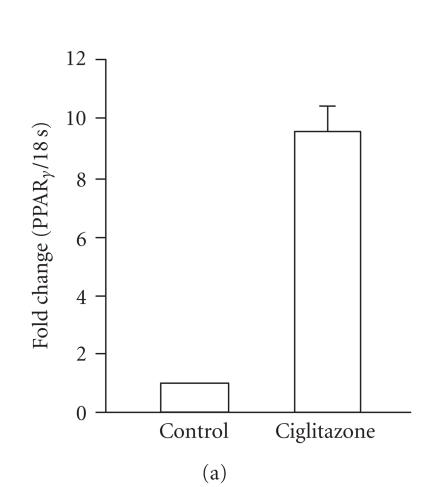

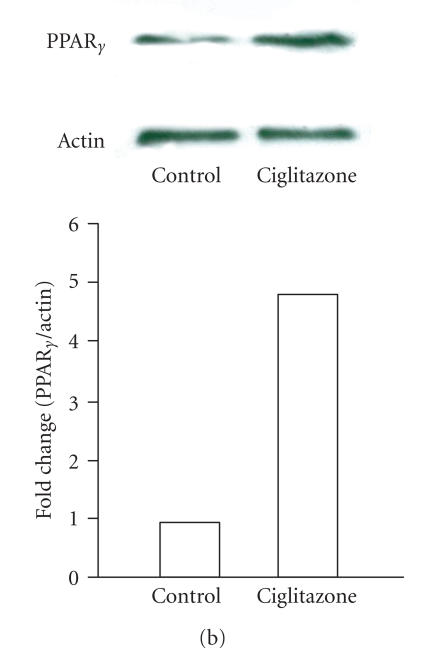
Ciglitazone promotes PPAR*γ* expression in the growth plate chondrocytes. Growth plate chondrocytes in pellet cultures were incubated in the presence or absence of 5 *μ*M of ciglitazone for 4 days. (a) Total RNA was collected and real-time PCR was performed to quantitate PPAR*γ* mRNA levels, which were normalized with respect to endogenous 18S rRNA levels. (b) Proteins were extracted for immunoblotting to detect PPAR*γ* expression and the immunoblots quantitated using Kodak 1D image analysis software. Actin was used as an internal control.

Treatment of growth plate cells with ciglitazone resulted in increases of both PPAR*γ* mRNA and protein. PPAR*γ* mRNA was increased 9-fold after addition of ciglitazone (5 *μ*M) for 4 days (Figure 1(a)), while PPAR*γ* protein levels were increased approximately 5-fold (Figure 1(b)).

### PPAR*γ* induces adipogenic differentiation in growth plate chondrocytes

**Figure 2:**
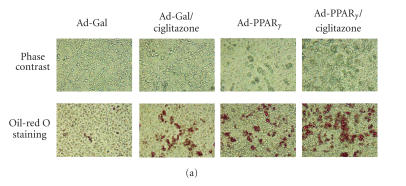

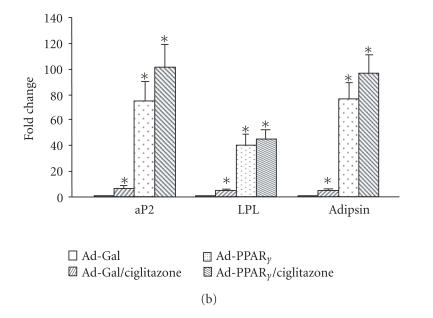
Adipogenic changes in the growth plate cells in which PPAR*γ* was overexpressed or cells treated with ciglitazone. (a) Growth plate cells were infected with Ad-PPAR*γ* or Ad-Gal followed by a 10-day incubation in the presence or absence of ciglitazone (5 *μ*M). Cells were observed under the phase contrast microscope with 10-fold magnification. Oil Red-O staining shows lipid accumulation within the cells. (b) Quantitative real-time PCR analysis of the adipogenic marker genes aP2, LPL, and adipsin, in growth plate cells at day 4 of the culture. Ad-Gal- infected cells were used as controls. Gene expression levels were normalized with respect to endogenous 18S rRNA. * *P* < .05 versus the expression in control cells.

Phase-contrast microscopy demonstrated that the Ad-PPAR*γ*-infected growth plate chondrocytes acquired the morphology characteristic of adipocytes after culture in monolayer for 10 days. Approximately 50% of the cells had accumulated vacuoles, which were positive for Oil Red-O staining of lipid accumulation (Figure 2(a)). The control cells infected with Ad-Gal demonstrated few Oil Red-O positive vacuoles. Cells treated with 5 *μ*M of ciglitazone alone for 10 days also showed enhanced Oil Red-O staining (20% of the total cells), while the combination of ciglitazone treatment and Ad-PPAR*γ* infection further enhanced lipid accumulation (over 70% of cells staining positively with Oil Red-O).

To characterize the phenotype of the transformed cells in more detail, the cells were cultured in three-dimensional cell pellets and the expression of adipocyte differentiation marker genes examined by real-time RT-PCR at day 4 of the culture period. Compared with the control samples, the levels of expression of the adipogenic marker genes aP2, LPL,and adipsin increased 6.6-, 4.4- and 4.6-folds, respectively, on day 4 in the 5 *μ*M ciglitazone-treated cells (Figure 2(b)). Expression of aP2, LPL, and adipsin genes increased in the Ad-PPAR*γ*-infected cells by 75.1-, 40.2-, and 76.6-folds, respectively in the absence of ciglitazone, and 101.1-, 44.9-, and 96.3-folds, respectively, in the presence of ciglitazone (5 *μ*M).

**Figure 3:**
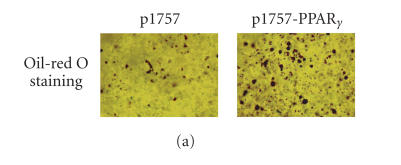

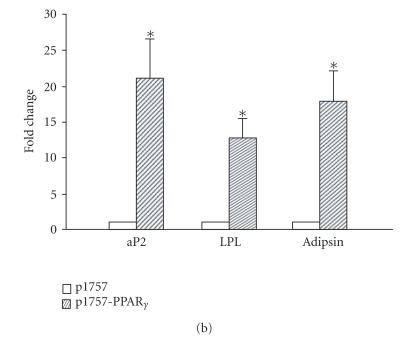
Adipogenic changes in the growth plate chondrocytes transfected with a PPAR*γ* plasmid under the control of a collagen *α*1(II) promoter. (a) Growth plate cells were transfected with the p1757-PPAR*γ* plasmid. Transfected cells were maintained in pellet cultures for 10 days, followed by Oil Red-O staining. (b) Quantitative PCR analysis of adipogenic markers of the p1757-PPAR*γ* transfected growth plate cells at day 4 after transfection. Cells transfected with the empty p1757 plasmid were used as controls. Gene expression levels were normalized with respect to endogenous 18S rRNA. * *P* < .05 versus the expression in control cells.

In order to address the possibility that PPAR*γ* was acting on an undifferentiated mesenchymal stem cell as opposed to a differentiated chondrocyte, p1757-PPAR*γ* expression plasmid was generated in which a PPAR*γ* cDNA was placed under the transcriptional control of the rat COL2A1 gene promoter and enhancer sequences. Oil Red-O staining of the p1757-PPAR*γ*-transfected cells maintained in three-dimensional pellet culture for 10 days showed markedly increased lipid accumulation (Figure 3(a)). Real-time RT-PCR demonstrated that aP2, LPL, and adipsin mRNA expressions were upregulated 21.1-, 12.9-, and 17.9-folds, respectively, compared with the cells transfected with the empty p1757 plasmid at day 4 after transfection (Figure 3(b)).

### PPAR*γ* induces loss of chondrocytic phenotype in growth plate cells

**Figure 4:**
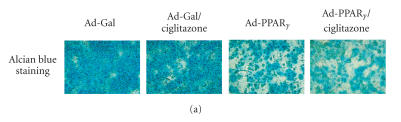

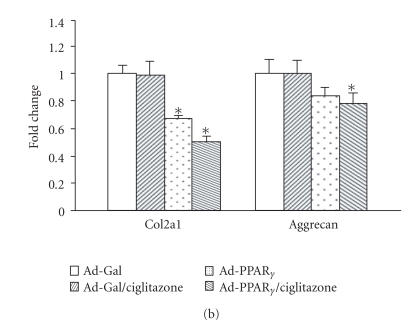
Loss of the chondrogenic phenotype in growth plate cells in which PPAR*γ* was overexpressed or cells treated with ciglitazone. (a) Growth plate cells were infected with Ad-PPAR*γ* or Ad-Gal followed by a 10-day incubation in the presence or absence of ciglitazone (5 *μ*M). Alcian blue staining shows the accumulation of chondrocyte-specific matrix. (b) Quantitative PCR analysis of chondrogenic genes, COL2A1, and aggrecan, in growth plate cells infected with Ad-PPAR*γ* or treated with ciglitazone at day 4 of treatment. Ad-Gal-infected cells were used as controls. Gene expression levels were normalized with respect to endogenous 18S rRNA. * *P* < .05 versus the expression in control cells.

Alcian blue staining was used to detect the accumulation of cartilage-specific proteoglycan. At day 10, the control cultures of growth plate cells still accumulated abundant proteoglycan (Figure 4(a)). No significant difference in proteoglycan accumulation was observed in the growth plate chondrocytes that were treated with 5 *μ*M of ciglitazone alone. Compared to the Ad-Gal-infected cells, the Ad-PPAR*γ*-infected cultures were stained less intensely with Alcian blue, especially the surrounding of the cells that contained vacuoles. Addition of 5 *μ*M of ciglitazone to the Ad-PPAR*γ*-infected cultures resulted in a further decrease in proteoglycan matrix.

Quantitative RT-PCR demonstrated that the chondro-cyte-specific genes COL2A1 and aggrecan were downregulated by both PPAR*γ* and ciglitazone (Figure 4(b)). Treatment with 5 *μ*M of ciglitazone for 4 days resulted in a 33% decrease of COL2A1 mRNA and a 17% decrease in aggrecan mRNA expression. Combination of both PPAR*γ* adenovirus and 5 *μ*M of ciglitazone resulted in a 50% decrease of COL2A1 mRNA and a 22% decrease in aggrecan mRNA expression.

### PPAR*γ* inhibits T3-induced hypertrophy and mineralization in growth plate chondrocytes

**Figure 5:**
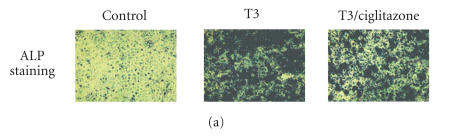

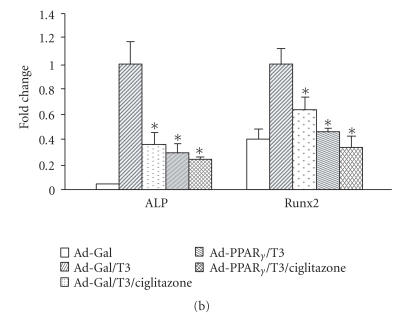
Inhibition of T3-induced hypertrophy and mineralization in the growth plate cells by PPAR*γ* overexpression or ciglitazone treatment. (a) Growth plate cells were treated with T3 (100 ng/mL) in the presence or absence of ciglitazone (5 *μ*M) for 10 days. Alkaline phosphatase (ALP) staining was used as a marker of terminal differentiation of growth plate chondrocytes. Positive stainings were colored in dark blue. Negative-stained background was colored in light green. (b) Quantitative PCR analysis of ALP and Runx2 genes 4 days after growth plate cells were infected with Ad-PPAR*γ* or treated with ciglitazone. Ad-Gal-infected cells without T3 or ciglitazone treatment were used as controls. Gene expression levels were normalized with respect to endogenous 18S rRNA. * *P* < .05 versus the expression in the Ad-Gal-infected cells with T3-treatment.

Thyroid hormone is a crucial regulator in growth plate chondrocyte hypertrophic differentiation and matrix mineralization [15–17]. Growth plate cells treated with thyroid hormone and 5 *μ*M of ciglitazone demonstrated decreased alkaline phosphatase histochemical staining compared to cells treated with T3 alone (Figure 5(a)). Quantitative RT-PCR analysis of growth plate cells in pellet cultures treated with T3 and 5 *μ*M of ciglitazone for 4 days confirmed a 64% decrease in ALP mRNA compared to cells treated with T3 alone (Figure 5(b)). Infection with PPAR*γ* adenovirus in cells treated with T3 also decreased expression of ALP mRNA approximately 71% in the absence of ciglitazone, and 76% in the presence of 5 *μ*M of ciglitazone. Runx2/Cbfa1 is expressed in chondrocytes as they initiate chondrocyte hypertrophy and maturation. Ciglitazone at a concentration of 5 *μ*M decreased the T3-induced expression of Runx2 by 36%. Ad-PPAR*γ* infection decreased the expression of Runx2 mRNA by 54% in the absence of ciglitazone and by 66% in the presence of 5 *μ*M of ciglitazone.

## DISCUSSION

Growth plate chondrocytes originate from multipotential mesenchymal stem cells that can differentiate into other cell types including adipocytes. We present evidence in this study that growth plate cells continue to display differentiation plasticity and are able to undergo adipogenic changes and a reciprocal decrease of chondrocytic markers when PPAR*γ* is overexpressed.

It has been previously reported that chondrocytes are able to transdifferentiate into adipocytes in vitro [9]. The fatty acid content of the serum added to the culture media has been implicated as a potential cause of this trans-differentiation process [6]. We used a serum-free culture system in these experiments to avoid the possibility that fatty acids in the serum might induce the adipogenic changes observed.

Ciglitazone is one of the thiazolidinedione classes of antidiabetic compounds which can activate PPAR*γ* [18]. Ciglitazone not only increases endogenous PPAR*γ* transcriptional activity [11], but also upregulates PPAR*γ* mRNA and protein expression in growth plate chondrocytes, as observed in this study.

Activation of endogenous PPAR*γ* by ciglitazone or adenoviral overexpression of PPAR*γ* in growth plate chondrocytes resulted in acquisition of adipogenic features in both high-density monolayer cultures and three-dimensional pellet cultures of growth plate chondrocytes, as evidenced by cell morphology, lipid accumulation, and expression of adipocyte marker genes aP2, LPL, and adipsin. Growth plate cells maintained in monolayer cultures seemed to acquire features of the adipocytic phenotype and lose features of the chondrocytic phenotype more readily than those in the pellet cultures (data not shown).

To confirm that the adipocyte-like cells were differentiated directly from chondrocytes and not from other cell types such as undifferentiated mesenchymal stem cells, growth plate cells were transfected with a PPAR*γ* plasmid under the control of a collagen *α*1(II) promoter. Acquisition of the adipogenic phenotype in these transfected cells was similar to the cells infected with an adenovirus encoding PPAR*γ* and driven by the CMV promoter/enhancer.

While PPAR*γ* and ciglitazone converted cells of the chondrocyte lineage to an adipocytic phenotype, features of the chondrocyte phenotype were simultaneously suppressed. PPAR*γ* inhibited the ability of chondrocytes to terminally differentiate into hypertrophic cells, and suppressed the expression of genes encoding chondrocyte-specific extracellular matrix proteins.

Slipped capital femoral epiphysis (SCFE) is an obesity-related hip disease in children characterized by weakness in the growth plate of the proximal femur, delayed skeletal maturation, and eventual mechanical failure of the physis [19–21]. We speculate that obesity may induce the expression of PPAR*γ* isoforms in growth plate chondrocytes, resulting in phenotypic changes that interrupt normal skeletal maturation at the growth plate through interference with thyroid hormone signaling. This interference with thyroid hormone-mediated terminal differentiation of growth plate cells and resulting decreased mineralization of the cartilage matrix would be expected to reduce the resistance of the growth plate to shear stresses. Therefore this delay in maturation at the growth plate, combined with both the increased mechanical stress resulting from increased body weight and the decreased shear stress resulting from delayed maturation, may combine to cause the proximal femoral epiphysis to slip.

## Figures and Tables

**Table 1:** Primer sequences used for real-time PCR.

Genes	Primers	

aP2	Forward	5′-GGCTTCGCCACCAGGAA-3′
	Reserve	5′-CCCTTCTACGCTGATGATCAAGT-3′
LPL	Forward	5′-GGGTCGCCTGGTCGAAGT-3′
	Reserve	5′-AAAGTGCCTCCATTGGGATAAA-3″
Adipsin	Forward	5′-CCGATGTCCTGCAGCAACT-3′
	Reserve	5′-CATGGTACGTGCGCAGATTG-3′
COL2A1	Forward	5′-GGTGGAGCAGCAAGAGCAA-3′
	Reserve	5′-CGTCGCCGTAGCTGAAGTG-3′
Aggrecan	Forward	5′-CTAGCTGCTTAGCAGGGATAACG-3′
	Reserve	5′-CCGCAGAGTCACAAAGACCAA-3′
ALP	Forward	5′-GCCGGCAGGACACAGACT-3′
	Reserve	5′-GGTTGCAGGGTCTGGAGAGTATA-3′
Runx2/	Forward	5′-TTTAGGGCGCATTCCTCATC-3′
Cbfa1	Reserve	5′-GGAGGGCCGTGGGTTCT-3′
18S	Forward	5′-AGTCCCTGCCCTTTGTACACA-3′
	Reserve	5′-GATCCGAGGGCCTCACTAAAC-3′
